# Parliament and Publications

**Published:** 1910-12-10

**Authors:** 


### Parliament and Publications.

### BARBADOS GENERAL HOSPITAL.

The Barbados General Hospital, the only hospital in the island, receives from the Colonial Government an annual grant of £6,630 for its upkeep. During last year 3,514 persons were treated in the hospital, of whom 310 died. The number of out-patients treated during the same period was 28,212, while the daily average in hospital was 210. There is no law in Barbados providing for the compulsory segregation of lepers, but on January 1, 1909, there were resident in the Lazaretto 117 inmates.—"Colonial Reports—Barbados," Cd. 4964, price 2½d.

### MEMORANDUM ON PLAGUE.

The Local Government Board has issued to local authorities a Memorandum on Plague, containing recommendations which may be summarised as follows: (1) Persistently and systematically destroy all rats; (2) remove and obliterate their nests, burrows, and habitual haunts; and (3) make each dwelling as far as practicable rat-proof, and remove all known harbourage for rats in or near dwellings; (4) at the same time do not allow waste food (whether for human beings, chickens, or other animals) to accumulate in or about the house. The Memorandum deals with the symptoms, diagnosis, and methods of spread of plague, precautions to be taken against infection, and the extermination of rats.

### OLD-AGE PENSIONERS AND MEDICAL RELIEF.

In reply to a question asked in the House of Commons, the President of the Local Government Board stated that there is nothing to prevent a person in receipt of an old-age pension applying to the Guardians for medical relief. He would, however, be disqualified under the Old Age Pensions Act for continuing to receive the pension while he was in receipt of ordinary poor relief. Certain relief is declared by law not to be a disqualification for registration as a parliamentary elector or a reason for depriving any person of any franchise, right, or privilege, and relief of this kind, which includes medical relief, would not constitute a disqualification for receiving an old-age pension or involve the loss of the franchise.

### THE TEACHING OF INFANT MANAGEMENT IN ELEMENTARY SCHOOLS.

In a Memorandum which has just been issued the Board of Education direct the attention of members of local education authorities, of managers, and of teachers in public elementary schools to the great importance of increasing and improving the present inadequate provision in the schools in England and Wales for instructing girls in the care and management of infants. A course for use in schools is suggested for girls between seven and twelve years of age, and another for girls between twelve and fourteen years of age. It is not desired to teach every schoolgirl a hotch-potch of semi-medical information on the various ailments and diseases to which infancy is liable, but to give her a simple and practical understanding of those things which make up a healthy home-life for little children. In the advanced course it is suggested that in the lessons on the care of infants, the dangers to child-life arising from unsuitable food, from the methods of feeding, from dirt in any form, from insufficient or inappropriate clothing, from cold or chill, from inadequate or disturbed sleep, from lack of air and exercise, etc., should be clearly pointed out.—"Board of Education Memorandum on the Teaching of Infant Care," Circular 758, price 2d.

### OVERLAPPING OF POOR-LAW AND SANITARY AUTHORITIES.

Mr. Henry Walker has asked the President of the Local Government Board whether persons suffering from certain diseases, notably phthisis, measles, and whooping cough, are being maintained and medically treated, free of charge, in some districts in institutions under the Poor Law and in others under the local sanitary authorities; and whether the Government propose to take any steps to prevent the overlapping and duplication of authorities in these cases, or to relieve any of the patients who may be entered and classed as paupers from being so designated. In a written reply Mr. Burns states that the Guardians are under an obligation to relieve destitution whenever application is made to them, and they are, therefore, primarily responsible for dealing with cases of the diseases mentioned, if the patients apply for relief and are in need of treatment which they are unable themselves to supply. Cases of the diseases mentioned are sometimes received by the sanitary authority, but the arrangement which the Local Government Board have systematically encouraged is that Guardians should enter into agreements with the sanitary authorities for the reception of infectious diseases into their hospitals. All cases chargeable to Boards of Guardians are entered in the return of paupers, but medical relief does not involve all the disabilities attaching to ordinary poor relief.

### THE HOSPITALS OF HONG KONG.

The Government Hospitals of Hong Kong consist of the Civil Hospital, the Victoria Hospital for Women and Children, and the Kennedy Town Infectious Diseases Hospital. There is an observation station capable of accommodating 1,500 persons in the event of an outbreak of infectious disease in a ship arriving in the harbour. The Civil Hospital contains 150 beds, and, according to a Government report just published, 2,384 in-patients and 16,981 out-patients were treated during 1909. Among Chinese hospitals the most important is the Tung Wah; this was opened in 1872, and is mainly supported by the voluntary subscriptions of Chinese, but receives an annual grant of 8,000 dollars from the Government. Only Chinese are treated in this institution, but Chinese as well as European methods of treatment are employed in accordance with the wishes expressed by the patients or their friends. About half the number are now treated by Western methods. The Hospital is managed by a committee of Chinese gentlemen annually elected, and is under the supervision of a visiting physician, while a Chinese house surgeon is a member of the hospital staff. Work on the Hospital for Chinese in the Kowloon Peninsula has commenced; it occupies a site of three acres, and will ultimately provide accommodation for two hundred and ten patients. When opened it will receive a grant of 8,500 dollars per annum from the Government. The general death-rate of Hong Kong last year was 21.68 per 1,000 among the Chinese community and 12.45 per 1,000 among the non-Chinese community, as compared with 28.35 and 14.78 respectively during 1908. Owing to the general improvement in the sanitary condition of the Colony and the simplification of dealing with plague-infected houses, it has been found possible to reduce the number of sanitary inspectors by the abolition of five special plague inspectors, whose duties are now performed by the district inspectors.—"Colonial Reports—Hong Kong," Cd. 4964-33, price 4d.

